# Detection and Molecular Characterization of Human Adenovirus Infections among Hospitalized Children with Acute Diarrhea in Shanghai, China, 2006–2011

**DOI:** 10.1155/2017/9304830

**Published:** 2017-12-28

**Authors:** Lijuan Lu, Huaqing Zhong, Liyun Su, Lingfeng Cao, Menghua Xu, Niuniu Dong, Jin Xu

**Affiliations:** Department of Clinical Laboratory, Children's Hospital of Fudan University, Shanghai 201102, China

## Abstract

*Background*: Human adenovirus (HAdV) is considered a significant enteropathogen associated with sporadic diarrhea in children. However, limited data are available regarding the epidemiology of HAdV in hospitalized children with viral diarrhea in Shanghai. The aim of this study was to characterize the epidemiology of HAdVs and describe their association with acute diarrhea in hospitalized children. *Methods*: A total of 674 fecal samples were subjected to PCR or RT-PCR to detect RVA, HuCV, HAstV, and HAdV. *Results*: HAdV infections were detected in 4.7% (32/674) of specimens, with detection rates of 13.4% (11/82), 4.6% (8/174), 3.2% (4/124), 4.1% (3/74), 2.0% (2/100), and 3.3% (4/120) from 2006 to 2011, respectively. Comprehensive detection of the four viruses revealed the presence of a high percentage (90.6%) of coinfections among HAdV-positive samples, where HAdV+RVA was the most prevalent coinfection. Of the 32 HAdV-positive samples, 50.0% (16/32) were classified as HAdV-41, and 18.8% (6/32) were classified as HAdV-3. Almost 94.0% of children infected with HAdV were less than 24 months of age. *Conclusions*: These results clearly indicated diversity across the HAdV genotypes detected in inpatient children with acute diarrhea in Shanghai and suggested that HAdVs play a role in children with acute diarrhea.

## 1. Introduction

Acute diarrhea is a major disease caused by various pathogenic bacteria, viruses, and parasites in all humans, but especially in children aged under 5 years. This condition remains a leading cause of morbidity and mortality worldwide, especially in developing areas. More than 50% of all diarrhea episodes have been found to be induced by viral pathogens [1]. Among the different kinds of diarrheal viruses, group A rotavirus (RVA) and human calicivirus (HuCV) have been identified as the major causes of acute diarrhea worldwide. Human adenovirus (HAdV) and human astrovirus (HAstV) have also been recognized as two additional primary causes of infectious diarrhea in pediatric patients [2, 3].

HAdVs are members of the genus *Mastadenovirus* in the family *Adenoviridae* and cause a wide spectrum of acute and chronic diseases, including acute diarrhea, respiratory illnesses, pneumonias, and pharyngoconjunctival fever [4]. HAdV is a linear, double-stranded DNA virus with a genome size of 26–45 kb. Following the development of phylogenetic and bioinformatic technologies, sequence-based typing strategies have been shown to serve as more rapid or sensitive methods than immunospecific methods for visualization via electron microscopy for HAdV detection. Over 70 HAdV genotypes in seven species (HAdV A–G) have been characterized and classified phylogenetically according to their nucleic acid characteristics and homologies as well as their hexon and fiber protein characteristics since HAdV was first isolated in 1953 [4, 5]. Among these species, HAdV-F types HAdV-40 and -41 have been found to be frequent causes of pediatric diarrhea and, as such, are known as enteric adenoviruses. Other adenoviruses are regarded as “nonenteric” adenoviruses. HAdV is not only considered a significant pathogen that occurs in association with sporadic acute diarrhea but is also a major enteropathogen responsible for nosocomial diarrhea in hospitals. HAdV diarrhea can be persistent and severe in immunocompromised hosts [6]. Thus, it is important to monitor the epidemiology of HAdV in hospitalized children with acute diarrhea.

Prior to this study, limited data were available regarding the epidemiology of HAdV in hospitalized children with viral diarrhea in Shanghai, and most studies have focused on RVA and HuCV infections [7–9]. Therefore, we conducted this study to evaluate the epidemiology of HAdV in hospitalized children in Shanghai upon the onset of viral diarrhea.

## 2. Materials and Methods

From January 2006 to December 2011, 674 stool samples were obtained from children under the age of 5 years with acute diarrhea enrolled at the Children's Hospital of Fudan University in Shanghai. All patients came from either the gastroenterology department, the infectious disease department, or the neonatal department. Each stool sample was collected from the patients when acute diarrhea was clinically suspected and stored at −70°C. Acute diarrhea cases were defined as three soft or liquid stools or three bouts of vomiting per 24 hours in a patient. When pus or blood was present in a sample, it was excluded, regardless of the identified fever conditions. Among the enrolled patients, 367 were male children, and 307 were female children. For subsequent data analysis, five different age groups were established: 0–12 months (504 samples), 13–24 months (104 samples), 25–36 months (29 samples), 37–48 months (27 samples), and 49–60 months (10 samples).

The fecal specimens were diluted to 10% suspensions with 0.9% physiological saline and then clarified via centrifugation at 8000 g for 10 min. The total RNA/DNA was extracted using the TIANamp Virus DNA/RNA Kit (Tiangen Biotech, Beijing, China) according to the manufacturer's instructions. The extracted viral RNA/DNA was dissolved in 40 *μ*L of nuclease-free water and stored at −70°C until analysis.

The extracted DNA was subjected to PCR amplification using primers specific to HAdV. A 482 bp fragment of a conserved region (C4) in the HAdV hexon gene (nt: 1834–2296) was amplified using the Ad-1 (5′-TTCCCCATGGCICAYAACAC-3′) and Ad-2 (5′-CCCTGGTAKCCRATRTTGTA-3′) primers [10]. The PCR cycling program was as follows: 94°C for 4 min, followed by 35 cycles of 94°C for 30 sec, 55°C for 30 sec, and 72°C for 1 min and a final extension cycle at 72°C for 7 min.

cDNA used for detecting HuCV and HAstV was synthesized using the extracted RNA and the PrimeScript™ RT Reagent Kit (Takara Bio Co., Dalian, China) according to the manufacturer's instructions. The obtained cDNA was used for the detection of HuCV and HAstV via PCR using specific primers, as previously described [9, 11].

The extracted RNA was also used as a template for the amplification of RVA via one-step reverse transcription PCR (RT-PCR) and seminested PCR with typing-specific primers, as reported in another paper [8].

Finally, all PCR products were electrophoresed in a 2% agarose gel with ethidium bromide and a DNA ladder of 100 bp (Takara Bio Co., Dalian, China).

The samples that were positive for HAdV, HuCV, and HAstV were subjected to nucleotide sequencing by the Shanghai Sunny Biotechnology Co., Ltd., and Sanger sequencing was carried out using the BigDye Terminator v3.1 Cycle Sequencing Kit (Thermo Fisher Scientific Inc., U.S.) according to the manufacturer's instructions. Phylogenetic analyses of the detected HAdVs were conducted using the MEGA version 6.0 software package. The phylogenetic tree was constructed using the Kimura two-parameter method. The reference HAdV strains and accession numbers used in this study were as follows: HAdV-1: AC_000017, AF534906, KC632723; HAdV-2: J01917; HAdV-3: AY599836, AY854176, KM458623, KX384958; HAdV-5: AY339865; HAdV-7: KM458626, KU361344, KC857700; HAdV-8: AB448767; HAdV-11: AY163756; HAdV-12: X73487; HAdV-14: AY803294; HAdV-16: AY601636; HAdV-17: AF108105; HAdV-18: GU191019; HAdV-21: KJ364591; HAdV-31: AB330112, AM749299, DQ149611; HAdV-34: AY737797; HAdV-35: AY128640; HAdV-37: AB448777, AB475144; HAdV-40: L19443, KU904311, AB330121, KU162869; HAdV-41: HQ326161, DQ315364, KF303070, KY316162; HAdV-49: DQ393829; HAdV-61: JF964962; HAdV-A: NC_001460; HAdV-B: NC_011202; HAdV-C: NC_001405; HAdV-D: AC_000006; HAdV-E: NC_003266; and HAdV-F: NC_001454.

## 3. Results

Thirty-two of the 674 (4.7%) children with acute diarrhea were infected with HAdV between 2006 and 2011. The prevalence rates of HAdV were 13.4% (11/82), 4.6% (8/174), 3.2% (4/124), 4.1% (3/74), 2.0% (2/100), and 3.3% (4/120) in 2006, 2007, 2008, 2009, 2010, and 2011, respectively. Of the 32 HAdV-infected cases, 29 (90.6%) were coinfected with other viruses, while only 3 (9.4%) cases consisted of monoinfections. The most frequently identified mixed infection was HAdV + RVA (28.1% of the 32 cases). We also detected 14 cases of triple gastrointestinal tract infections, including the combinations HAdV + RVA + HAstV (25% of the 32 cases) and HAdV + RVA + HuCV (18.8% of the 32 cases). All four of the viruses (HAdV, RVA, HuCV, and HAstV) were detected simultaneously in 5 cases.

**Figure 1:**
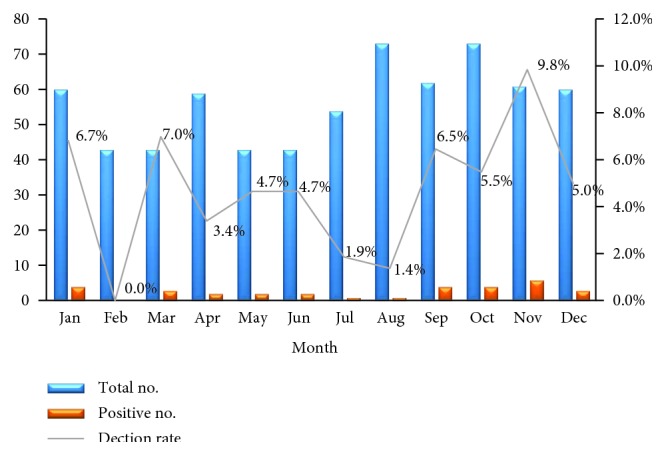
Monthly distribution of HAdV in hospitalized children with acute diarrhea in Shanghai between January 2006 and December 2011.

The seasonal distribution of HAdV infections between 2006 and 2011 is shown in Figure 1. The HAdV detection rate presented distinct seasonal variation, with a higher detection rate identified in autumn and winter months.

**Figure 2:**
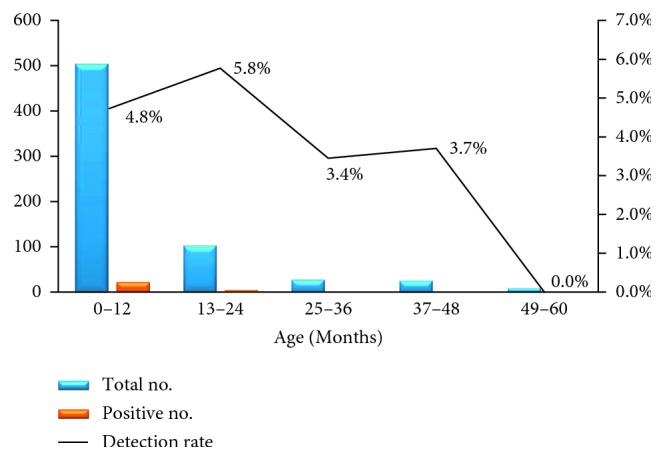
Age distribution of HAdV infection among hospitalized children with acute diarrhea in Shanghai between January 2006 and December 2011.

In this study, 93.8% of children infected with HAdV were aged less than 24 months, and the highest detection rate was found in children between 13 and 24 months of age (6 of 104). Overall, HAdV infections were only detected in children less than 48 months of age (Figure 2).

**Figure 3:**
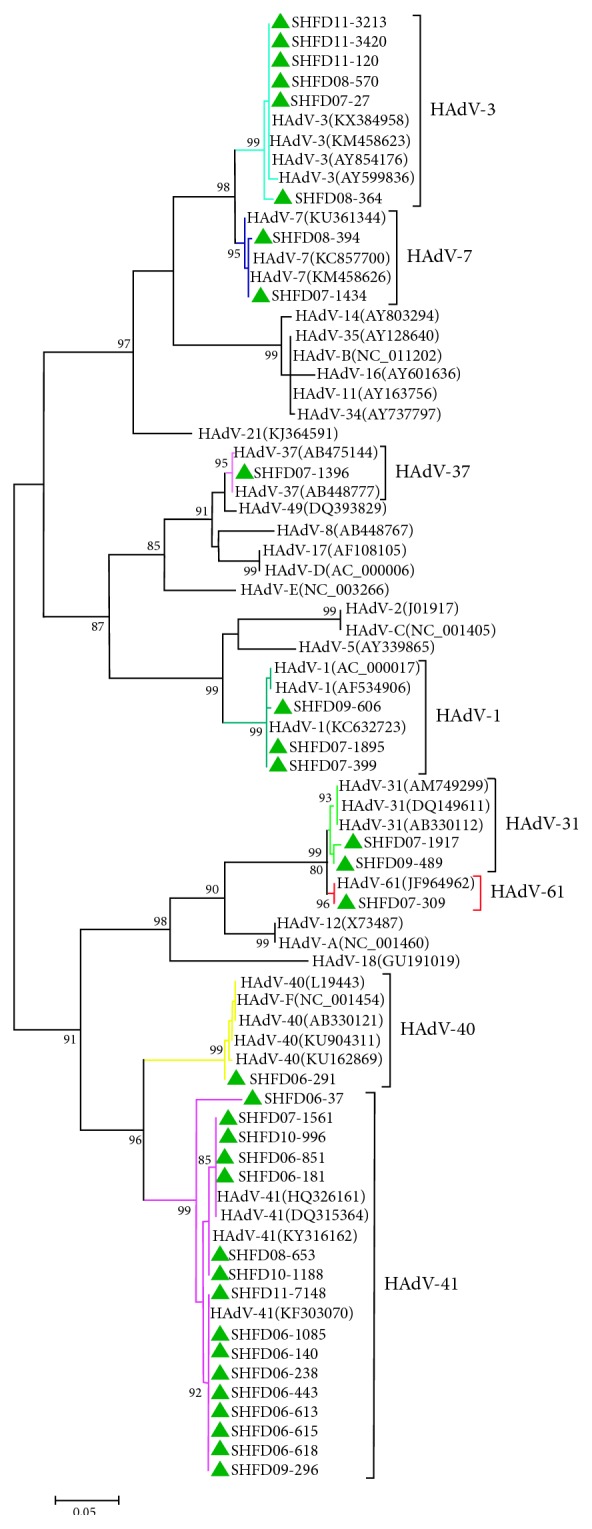
Phylogenetic tree based on partial nucleotide sequences (482 bp) of the hexon gene of HAdVs detected in hospitalized children with acute diarrhea in Shanghai, China, between January 2006 and December 2011. The delta symbol indicates the adenovirus strains detected in this study.

A total of 32 HAdV sequences were phylogenetically analyzed based on the hexon gene-based classification scheme using MEGA 5.0. The phylogenetic tree analysis showed clear predominance of HAdV-41 (16 of 32), followed by HAdV-3 (6 of 32), whereas only one case of infection with HAdV-40 was detected from 2006 to 2011. Distinct differences in the prevalence of HAdV genotypes were observed by year. In 2006, 90.9% of HAdV-positive samples corresponded to HAdV-41, and only one HAdV-41 strain was detected. Various HAdV genotypes were detected in 2007, including HAdV-1, -3, -7, -31, -37, -41, and -61. The predominant HAdV genotype identified in 2008 and 2011 was HAdV-3. The HAdV-1, -31, and -41 genotypes of HAdV were detected in 2009. Finally, only two HAdV-41 strains were detected in 2010 (Figure 3 and Table 1).

## 4. Discussion

Prior to this study, no research aimed at determining the burden of HAdV-related diarrhea among children in Shanghai had been performed. We conducted this study to assess the infection status and HAdV distribution patterns of hospitalized children with acute diarrhea in Shanghai from January 2006 to December 2011. The proportion of participating children with acute diarrhea in whom HAdV was detected was 4.7%, which was similar to previously reported rates in most studies conducted in Bangladesh, South Korea, Vietnam, and some other areas of China [12–19]. During the study period, the highest HAdV detection rate was observed in 2006, while the rate was lower than 5.0% in the remaining years. There was no evidence that HAdV led to an epidemic in 2006, as all of the HAdV-positive samples were distributed in 8 months (data not shown). The reasons for the decline observed from 2006 to 2011 may be improved environmental hygiene conditions and increased hygiene awareness. Similar to other studies [2, 3, 15, 20, 21], we found that most HAdV infections were detected in conjunction with RVA, HuCV, or HAstV, while monoinfection with HAdV alone was identified in only three samples. Additional research should be conducted in the future to determine whether coinfection with HAdV and other viruses serves as a characteristic feature of HAdV infection.

Our study also revealed that HAdV infections occurred throughout the year and peaked in the autumn and winter. This finding was in accordance with that of other studies conducted in Qingdao city and Hunan Province in China [22, 23]. However, in Tianjin, a northern city in China, HAdV infections are concentrated during the summer [16]. Although the reason for this difference remains unknown, more attention should be paid to the epidemiology of HAdV in Shanghai during different seasons to prevent HAdV outbreaks in hospitals.

In line with the findings of other studies [15–17], the majority of children with HAdV included in the present study were less than 2 years old. Similar trends have been detected in Tanzania and, in the case of the other three diarrhea viruses (RV, HuCV, and HAstV), in our previous studies [9, 24–26]. The reason that diarrhea-associated viral infections are much more likely to occur in younger children may be the immature immune function of infants and the fact that infections during infancy might result in protective immunity against reinfection at an older age [27, 28]. Furthermore, the high susceptibility of infants to pathogens may be influenced by differences in the intestinal microbiome, which changes over the first few of years of life [29–31].

According to the phylogenetic tree analysis conducted based on a partial genomic region of hexon, not only were species F (HAdV-40 and HAdV-41) strains detected, but other “nonenteric” HAdVs, such as species B (HAdV-3), were also detected in our study. However, we failed to perform an analysis of the temporal relationship between HAdV cases, as the reference strains used in this study were prototype strains rather than epidemic strains from different areas and times. The molecular characterization of these HAdV strains showed a clear predominance of HAdV-41 (50.0%), which was in agreement with the results of studies including children from Bangladesh, other Asian countries, and Sweden [13, 16, 32, 33]. However, in Dhaka city, Bangladesh, HAdV-40 was found to dominate other genotypes, whereas only one patient was infected with HAdV-40 in our study [13]. The reason for this disparity may be regional divergence leading to a higher prevalence of HAdV-41 than HAdV-40. Fluctuations in the prevalence of HAdV-40 and HAdV-41 have also been observed in other studies [34, 35]. Although some studies have shown that the duration of diarrhea is longer in patients with HAdV-41 infection than in those with HAdV-40 infection [32], it was nearly impossible to compare the duration of diarrhea between patients infected with HAdV-41 and HAdV-40 strains in this study because of the limited number of HAdV-40-positive samples.

In this study, one HAdV-61 strain belonging to the species A adenoviruses was detected in 2007. HAdV-61, resulting from an intraspecies recombination event between HAdV-12 and HAdV-31, was first isolated from a patient with acute diarrhea in Japan [36, 37]. The present study provides the first report of the epidemiology of HAdV-61 in China. Although this genotype has not expanded to show widespread prevalence in humans since it was discovered in 2004, more extensive research must be conducted in the future to prevent outbreaks. In addition, further studies are essential for understanding the full mechanisms of recombination in HAdV. We also detected two strains of HAdV-31, which is another enteric adenovirus that was found in association with diarrhea in an earlier study [38].

Interestingly, HAdV-3, the second leading HAdV genotype detected in the children included in our study (18.8%), is usually regarded as a pathogen that causes acute febrile pharyngitis, pharyngoconjunctival fever, acute respiratory disease, and even gastrointestinal symptoms [39]. A similarly high incidence of HAdV-3 was detected in outpatients in Korea and in a previous study conducted by our group in patients diagnosed with community-acquired diarrhea [35, 40]. According to the medical history of the patients included in our study, two patients exhibited monoinfections and were diagnosed only with diarrhea. In addition to HAdV-3, other “nonenteric” HAdV strains (HAdV-1, -7, and -37) were also detected in these hospitalized children with diarrhea. Moreover, a similar situation regarding the prevalence of “nonenteric” HAdVs (e.g., HAdV-1, -2, -3, -5, -6, and -8) has been found to occur in children with diarrhea in many other regions [13, 16, 38, 40]. As it has been established that respiratory types of HAdVs, such as HAdV-1, -2, and -3, can be shed in the feces of an infected person for months after the initial infection when these HAdVs infect respiratory sites [41, 42], the role of these “nonenteric” HAdVs in the onset of children's diarrhea requires further research.

## 5. Conclusions

In conclusion, this is the first report of the burden of HAdV-related diarrhea in Shanghai among hospitalized children younger than 5 years of age during the period from 2006 to 2011. The results of the present study showed that an increasing number of HAdV genotypes were detected in children with acute diarrhea. The rate at which HAdV was detected in conjunction with other diarrhea-causing viruses was relatively high in our study. Together, these findings underscore the importance of monitoring the epidemiology of HAdV infection and protecting vulnerable patients as part of the suite of infection prevention strategies in hospitals.

## Figures and Tables

**Table 1:** Distribution of HAdV genotypes in hospitalized children with acute diarrhea from 2006 to 2011.

Genotype	No. of HAdV-positive samples						
	2006 (*N* = 82)	2007 (*N* = 174)	2008 (*N* = 124)	2009 (*N* = 74)	2010 (*N* = 100)	2011 (*N* = 120)	Total (*N* = 674)
HAdV-1	0	2	0	1	0	0	3
HAdV-3	0	1	2	0	0	3	6
HAdV-7	0	1	1	0	0	0	2
HAdV-31	0	1	0	1	0	0	2
HAdV-37	0	1	0	0	0	0	1
HAdV-40	1	0	0	0	0	0	1
HAdV-41	10	1	1	1	2	1	16
HAdV-61	0	1	0	0	0	0	1
Total	11	8	4	3	2	4	32

*N*: total number tested each year.
